# Hot Deformation Behavior and Workability of In-Situ TiB_2_/7050Al Composites Fabricated by Powder Metallurgy

**DOI:** 10.3390/ma13235319

**Published:** 2020-11-24

**Authors:** Haofei Zhu, Jun Liu, Yi Wu, Qing Zhang, Qiwei Shi, Zhe Chen, Lei Wang, Fengguo Zhang, Haowei Wang

**Affiliations:** 1 State Key Laboratory of Metal Matrix Composites, Shanghai Jiao Tong University, Shanghai 200240, China; zhuhaofei183@sjtu.edu.cn (H.Z.); zhangqing0904@sjtu.edu.cn (Q.Z.); sqw@sjtu.edu.cn (Q.S.); leiwang798@sjtu.edu.cn (L.W.); hwwang@sjtu.edu.cn (H.W.); 2 School of Materials Science and Engineering, Shanghai Jiao Tong University, Shanghai 200240, China; eagle51@sjtu.edu.cn (Y.W.); fg.zhang@sjtu.edu.cn (F.Z.)

**Keywords:** aluminum matrix composites, powder metallurgy, thermo-mechanical deformation, microstructure, superplasticity

## Abstract

Isothermal compression tests of in-situ TiB_2_/7050Al composites fabricated by powder metallurgy were performed at 300–460 °C with the strain rate varying from 0.001 s^−1^ to 1 s^−1^. The Arrhenius constitutive equation and hot processing map of composites were established, presenting excellent hot workability with low activation energies and broad processing windows. Dramatic discontinuous/continuous dynamic recrystallization (DDRX/CDRX) and grain boundary sliding (GBS) take place in composites during deformation, depending on the Zener-Hollomon parameter (Z) values. It is found that initially uniform TiB_2_ particles and fine grain structures are beneficial to the DDRX, which is the major softening mechanism in composites at high Z values. With the Z value decreasing, dynamic recovery and CDRX around particles are enhanced, preventing the occurrence of DDRX. In addition, fine grain structures in composites are stable at elevated temperature thanks to the pinning of dense nanoparticles, which triggers the occurrence of GBS and ensures good workability at low Z values.

## 1. Introduction

Particle-reinforced aluminum matrix composites (PRAMCs) exhibit high elastic modulus and outstanding mechanical properties, which have been widely applied in aerospace, electronics and automotive industries over the past decades [1,2]. Generally, thermo-mechanical processing procedures such as hot rolling, forging and extrusion are necessary for PRAMCs in industrial application. These procedures can not only minimize defects and determine the dimensional accuracy, but also tailor the microstructures to optimize the mechanical properties of final products [3,4]. Therefore, lots of studies have focused on the thermal deformation behavior and associated microstructure evolution of PRAMCs.

Many research works revealed that the effects of particulates on deformation behavior of PRAMCs were complex [5]. Firstly, particles can hinder the motion of dislocations and grain boundary sliding (GBS) during deformation, leading to the accumulation of high-density dislocations around particles. Thus, the non-deformable particles can cause inhomogeneous strain distribution during plastic deformation [6,7,8,9,10]. Secondly, the particles could result in deformation instability (such as interface cracking, particle debonding and breakage) once the local stress near the interface exceeds the bonding strength of particles and matrices [11]. For instance, Li et al. [12] reported that voids, at low temperatures and high strain rates, tended to be formed in the vicinity of interfaces between B_4_C particles and Al matrices. Thirdly, particle deformation zones with high stored energy are ideal sites for the recrystallization nucleus (this mechanism is widely known as particle-stimulated nucleation (PSN)), which promotes the local discontinuous dynamic recrystallization (DDRX) during thermo-mechanical processes [6,13].

On the other hand, initial microstructures play a crucial role in the deformation behavior and microstructure evolution of PRAMCs as well [14]. For instance, Yang et al. [15] demonstrated that materials with fine grain structures exhibited lower flow stress and narrower instability deformation zones than those with coarse structures. Ren et al. [16] reported that fine-grained metal materials had favorable coordinated deformation capability and massive dynamic recrystallization (DRX) during the thermal deformation. Thus, due to the complex influence of particles and initial microstructure on hot deformation behavior, various deformation behaviors, including work hardening, dynamic softening or plastic instability, concurrently occur in PRAMCs [17,18]. Concentrating on the above deformation behaviors and underlying deformation mechanisms of composites during thermo-mechanical processing will help us to optimize processing parameters and prepare high-performance PRAMCs [19,20].

At present, there are a lot of literatures [21,22] about the deformation behavior and hot workability of PRAMCs fabricated by casting. It is inevitable that particles clusters formed by direct casting will accelerate the process of crack nucleation and propagation during deformation [23,24]. In contrast to PRAMCs prepared by casting, in-situ PRAMCs fabricated by powder metallurgy (PM) in this study initially consist of fine grain structure, dispersed particles and strong interface bonding strength, which have an important impact on the high-temperature deformation behavior and safe processing windows [25]. Therefore, it is necessary to undertake a systematic investigation on the hot deformation behavior and workability of in-situ PRAMCs fabricated by PM.

In this report, the constitutive equation with activation energy and hot processing map are established to evaluate the hot deformation behavior and workability of in-situ TiB_2_/7050Al composites fabricated by PM. The microstructure evolution and relevant deformation mechanisms of this composite are investigated in terms of Zener-Hollomon parameters, and the relationships between the initial microstructures and workability are discussed in detail.

## 2. Experimental Procedure

**Figure 1:**
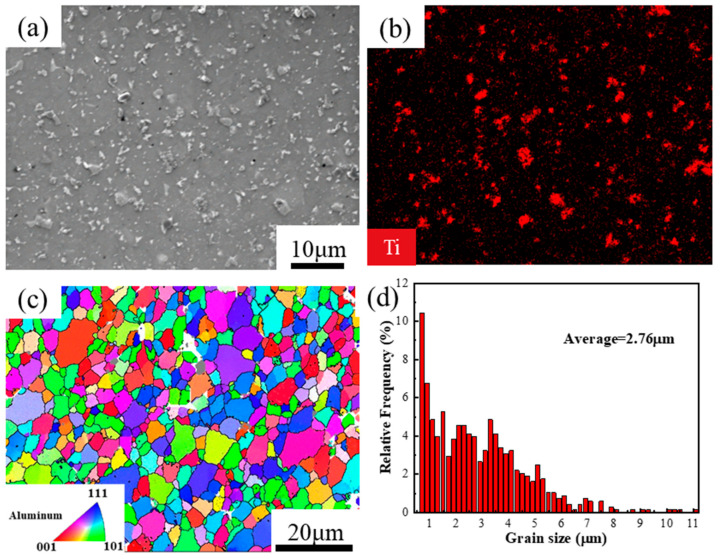
The initial microstructures of the powder metallurgy (PM) composites by scanning electron microscopy (SEM) and electron back-scattered diffraction (EBSD): (**a**) SEM morphology map, (**b**) Ti element distribution of (**a**), (**c**) EBSD map (high-angle grain boundaries (HAGBs) are in black lines, the gray phases are TiB_2_ particles, the white color regions are zero-solutions regions), (**d**) the grain size distribution of (**c**).

In-situ TiB_2_/7050Al composite powders (Institute of Special Materials, Shanghai Jiao Tong University, Shanghai, China) with chemical compositions of Al-6.30% Zn-2.18% Mg-2.20% Cu-6% TiB_2_ (wt %) were fabricated by an in-situ salt melt reaction and subsequently gas atomization, as described in [26,27]. The composite powders were consolidated by hot isostatic pressing (HIP) at 470°C for 3 h with a pressure of 130 MPa. Figure 1 shows the initial microstructures of in-situ TiB_2_/7050 Al composites fabricated by powder metallurgy (PM composites). It can be found that the TiB_2_ particles distribute uniformly in Al matrix (Figure 1a,b). Grain structures of composites after HIP are equiaxed and randomly orientated with an average grain size of 2.76 μm, as shown in Figure 1c,d.

Cylindrical specimens (Φ 10 mm × 15 mm) for hot isothermal compression tests were cut from the sintered TiB_2_/7050Al composite billets. The isothermal compression tests were carried out on the Gleeble-3180 thermo-mechanical simulator (DSI, St. Paul, MN, USA) at the temperatures of 300–460 °C and strain rates between 0.001 s^−1^ and 1 s^−1^. Before each test, the sample was heated to the experimental temperature at a rate of 10 °C/s and held for 5 min to ensure the uniform temperature throughout the sample. All the samples were compressed by constant stain rates to a true strain of 0.8, and the as-compressed samples were immediately quenched in water to retain the deformed microstructures.

Microstructure was characterized by a scanning electron microscope (SEM) (TESCAN MAIA3, Tescan, Brno, Czech Republic) and equipped electron back-scattered diffraction (EBSD) detector (BRUKER e-FlashHR, Bruker, Karlsruhe, Germany) with a step size of 0.14 μm. The data obtained were analyzed using the HKL Channel 5 software. The misorientation criterion for identifying high-angle grain boundaries (HAGBs) is larger than 15°, and the low-angle grain boundaries (LAGBs) refer to misorientation angles between 2° and 15°.

## 3. Results

### 3.1. Deformation Flow Behavior

**Figure 2:**
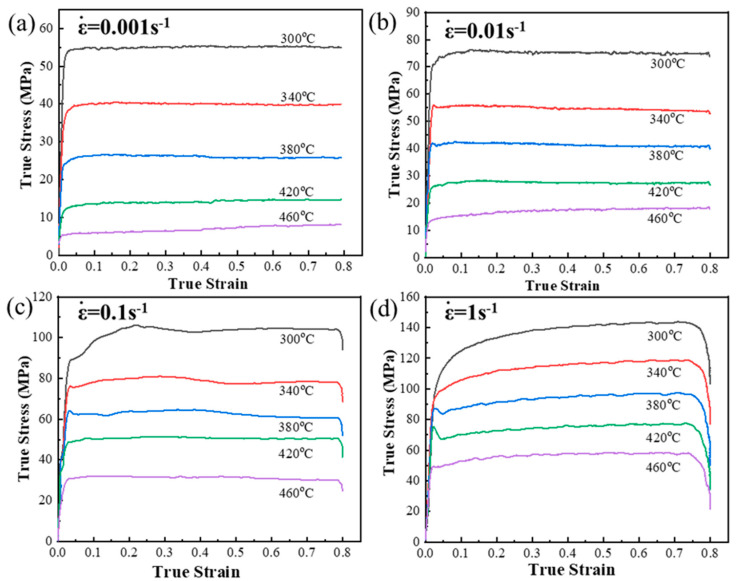
The true stress-strain curves of PM composites with different strain rates: (**a**) 0.001 s^−1^, (**b**) 0.01 s^−1^, (**c**) 0.1 s^−1^, (**d**) 1 s^−1^.

Figure 2 shows a series of true stress-strain curves of the PM composites at the deformation temperatures 300, 340, 380, 420, 460°C and the strain rates 0.001, 0.01, 0.1, 1 s^−1^, correspondingly. Obviously, the peak flow stress decreases with increasing the deformation temperature. Under all deformation parameters, the flow stress increase to yield strength values steeply in the initial compression stage. After that, the flow stress-strain curves exhibit four various configurations with the strains owing to the competition between strain hardening and dynamic softening [28]. (i) At high strain rate (1 s^−1^) and all temperatures (e.g., 300 °C/1 s^−1^), the flow curves display a continuously increasing type. (ii) At moderate strain rate (0.1 s^−1^) and low temperatures (e.g., 340 °C/0.1 s^−1^), the curves exhibit a transformative type that the flow stress firstly increases to peak stress and subsequently decreases to steady values. (iii) At low strain rates (0.01 s^−1^, 0.001 s^−1^) and moderate temperatures (e.g., 380 °C/0.01 s^−1^), the flow stress-strain curves exhibit a stable or slightly decreasing type. (iv) At low strain rates and high temperatures (e.g., 460 °C/0.001 s^−1^), the flow stress is very low and increases with strain slowly.

### 3.2. Constitutive Equation

To further understand the flow behavior and deformation mechanisms of the PM composites, constitutive equation and related activation energy are calculated, respectively. The constitutive equations based on the Arrhenius model can be used with precision to describe the relationship between flow stress, strain rate and temperature in the deformation process, which conduce to conduct thermo-mechanical behavior [29]. The constitutive equations containing the Zener-Hollomon parameter (Z) can be represented by:

(1) $$Z=\overset{\cdot }{\varepsilon }\exp\left(\frac{Q}{RT}\right)=A{\left[\sinh\left(\alpha \sigma \right)\right]}^{n}$$

(2) $$\overset{\cdot }{\varepsilon }=A_{1}\sigma^{n_{1}}\exp\left(-\frac{Q}{RT}\right);\;\alpha \sigma <0.8$$

(3) $$\overset{\cdot }{\varepsilon }=A_{2}\exp(\beta \sigma )\exp\left(-\frac{Q}{RT}\right);\;\alpha \sigma >1.2$$

(4) $$\overset{\cdot }{\varepsilon }=A{\left[\sinh\left(\alpha \sigma \right)\right]}^{n}\exp\left(-\frac{Q}{RT}\right);\;\mathrm{for}\;\mathrm{all}\;\sigma$$

where $\overset{\cdot }{\varepsilon }$ is the strain rate (s^−1^), *T* is the deformation temperature (K), *Q* is the deformation activation energy (J mol^−1^) and *σ* is the flow stress (MPa). And the R is gas constant (8.314 J mol^−1^ K^−1^) and A, A_1_, A_2_, n_1_, n, α, β (β = αn_1_) are material constants. It is well accepted that the exponential law of Equation (2) and the power law of Equation (3) can be used in low stress level and high stress level, respectively. The hyperbolic sine type law of Equation (4) covers a wide range of stress.

By taking the logarithm for both sides of Equations (1)–(4), the relations are expressed as:

(5) lnZ=lnA+nln[sinh(ασ)]

(6) $$\ln\overset{\cdot }{\varepsilon }=\ln A_{1}+n_{1}\ln\sigma -\frac{Q}{RT}$$

(7) $$\ln\overset{\cdot }{\varepsilon }=\ln A_{2}+\beta \sigma -\frac{Q}{RT}$$

(8) $$\ln\overset{\cdot }{\varepsilon }=\ln A+n\ln\left[\sinh\left(\alpha \sigma \right)\right]-\frac{Q}{RT}$$

By fixing the temperature or strain rate, and taking the partial differential of Equation (8), the *Q* can be represented:

(9) Q=R[∂lnε·∂ln[sinh(ασ)]]T[∂ln[sinh(ασ)]∂(1T)]ε·

**Figure 3:**
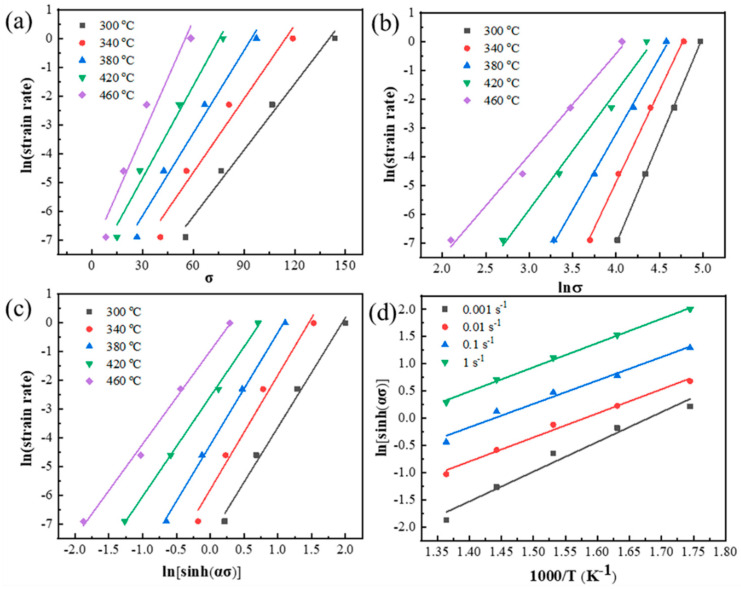
The correlations of (**a**) ln$\overset{\cdot }{\varepsilon }$-*σ_p_*, (**b**) ln$\overset{\cdot }{\varepsilon }$-ln*σ_p_*, (**c**) ln$\overset{\cdot }{\varepsilon }$-ln[sinh(α*σ_p_*)], (**d**) ln[sinh(α*σ_p_*)]-1000/*T*.

**Figure 4:**
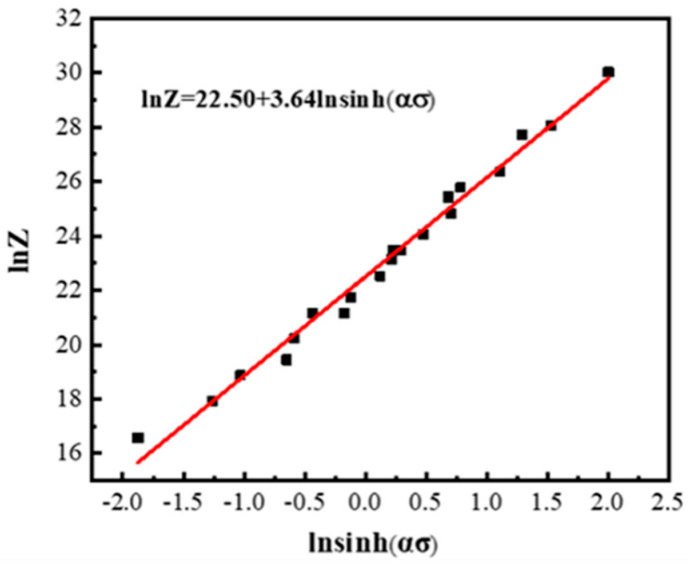
The correlations between lnZ and ln[sinh(α*σ*)].

In order to guarantee the safe operation of equipment under the rated maximum load, the peak stress *σ_p_* is chosen to determine the material constants in Equations (1)–(9). Figure 3 shows the correlations of ln$\overset{\cdot }{\varepsilon }$-*σ_p_*, ln$\overset{\cdot }{\varepsilon }$-ln*σ_p_*, ln$\overset{\cdot }{\varepsilon }$-ln[sinh(α*σ_p_*)] and ln[sinh(α*σ_p_*)]-1000/T. The values of n_1_, β, and *Q* can be acquired from the average slope of fitting lines in Figure 3. Figure 4 represents the correlation of lnZ-ln[sinh(α*σ_p_*)]. Through linear regression analysis, the slope and intercept of fitting line in Figure 4 are the parameters n and lnA, respectively. The values of n_1_, β, α, *Q*, n and lnA are calculated as shown in Table 1. Therefore, the Arrhenius constitutive equation based on Equation (1) is established as follows:

(10) σp=10.0212ln{(Z5.93×109)13.64+[(Z5.93×109)23.64+1]12}

It is widely known that the value of activation energy *Q* is closely related to the thermodynamic mechanism of dislocation motion [18]. In previous reports [13,22,30], the PRAMCs usually exhibit higher activation energy values than the lattice diffusion of aluminum (142 kJ mol^−1^) because of the dragging forces of particles. However, the average value of *Q* in this work is 143.11 kJ mol^−1^ approximately to 142 kJ mol^−1^, which suggests that thermal deformation of this composites is dominantly controlled by the cross-slip or climbing of dislocations [31]. In addition, the value of *Q* can reflect the hot workability of deformed materials, and the improvement of workability will occur in materials with low activation energy [18]. Compared to other PRAMCs [13,22,30], the PM composites with lower value of *Q* may have better hot workability and the detailed analyses of workability are given in the following sections.

### 3.3. Processing Map

**Figure 5:**
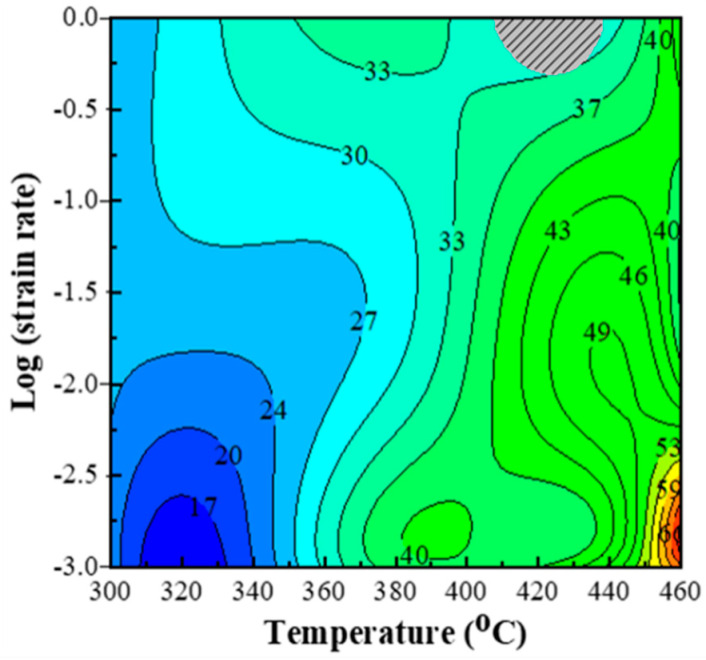
Processing map of the PM composites at true strain of 0.8 (the gray-shadowed areas represent the flow instability).

The processing map based on the dynamic material model (DMM, the details are given in Appendix A) [32] has been proved and widely accepted as an intuitive display for the response of materials to deformation conditions. It has been generally used to determine the optimal strain rates and temperatures for secondary processing [33]. Figure 5 illustrates the hot processing map of PM composites at the true strain of 0.8. The contour values show the power dissipation efficiency (*η*) that illustrates the degree of power dissipation. Generally, materials deformation with the high value of *η* will have preferable workability. The efficiency value *η* is thought to be closely related to the microstructure evolution, such as dynamic recovery (DRV), DRX and superplastic flow. It was reported that [17,22,34] the value of *η* in the DRV dominating region was about 20–30% and the efficiency in the DRX controlling region should be in range of 30–50%. When the power dissipation efficiency is high, up to 60–70%, superplastic deformation will occur.

At the overall trend in the processing map (Figure 5), the efficiency *η* value increases gradually with deformation temperature increasing or strain rate decreasing. The region of instability is narrow and only locates in the temperature range of 405–440 °C within the strain rate range of 0.5–1 s^−1^. Most of *η* values in the safe working domain are larger than 20% and the peak value is high up to 69% at 460 °C/0.001 s^−1^. In our previous work [35], the instability zone of the in-situ TiB_2_/7050Al matrix composites fabricated by direct-chill casting dominated in the strain rate from 0.1 s^−1^ to 1 s^−1^ and the temperature from 300 °C to 400 °C. The maximum *η* value was 36%. Park et al. [17] reported that the instability region in processing maps of as-cast 7075 Al alloys was located in 300–350 °C/0.1–10 s^−1^ and the maximum value of *η* was 39%. Wu et al. [36] reported that the SiC/7075Al composites produced by spray deposition had two instable domains in the processing maps around 300–390 °C/0.05–1 s^−1^ and 400–450 °C/0.05–1 s^−1^, respectively. Compared to the previous reports, the PM composites exhibit excellent hot workability.

### 3.4. Microstructure Evolution with Zener-Hollomon Parameters

**Figure 6:**
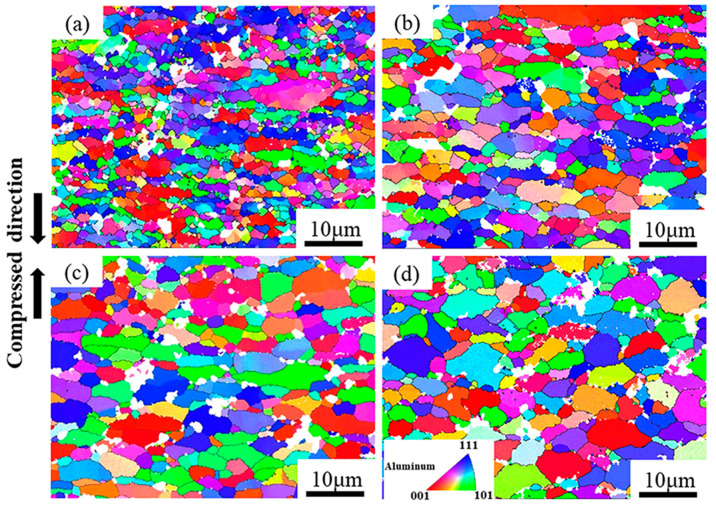
The inverse pole figures (IPF) maps of PM composites under different Z values: (**a**) lnZ = 30.0 (300 °C/1 s^−1^), (**b**) lnZ = 25.8 (340 °C /0.1 s^−1^), (**c**) lnZ = 21.7 (380 °C/0.01 s^−1^), (**d**) lnZ = 16.6 (460 °C /0.001 s^−1^) (HAGBs are in black lines).

**Figure 7:**
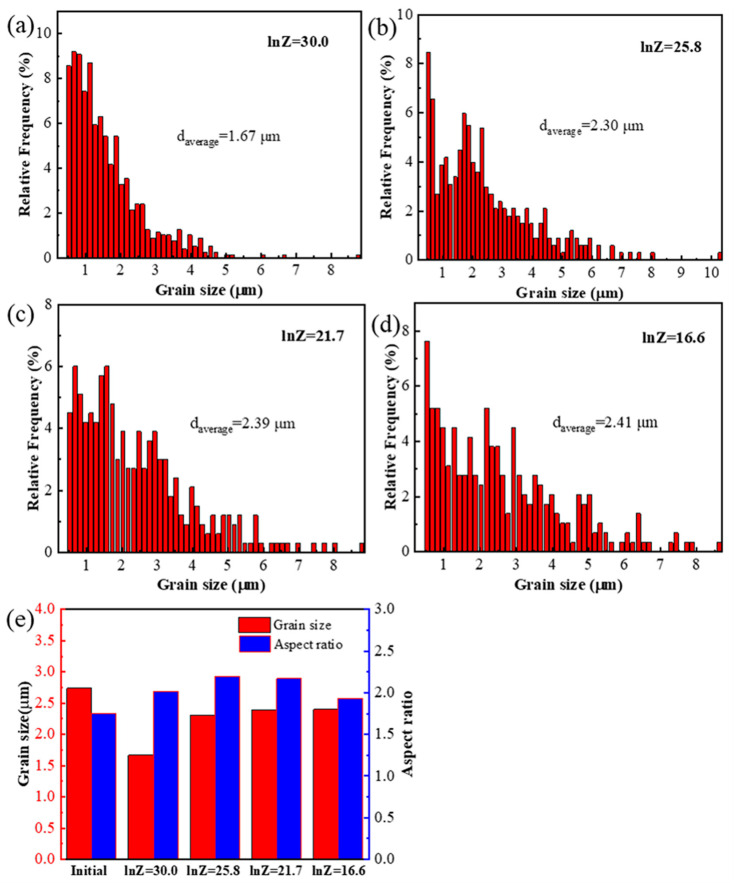
The grain size distribution of deformed samples under different Z parameters: (**a**) lnZ = 30.0, (**b**) lnZ = 25.8, (**c**) lnZ = 21.7, (**d**) lnZ = 16.6; (**e**) the comparison of average grain size and aspect ratio between deformed composites and initial microstructure.

**Figure 8:**
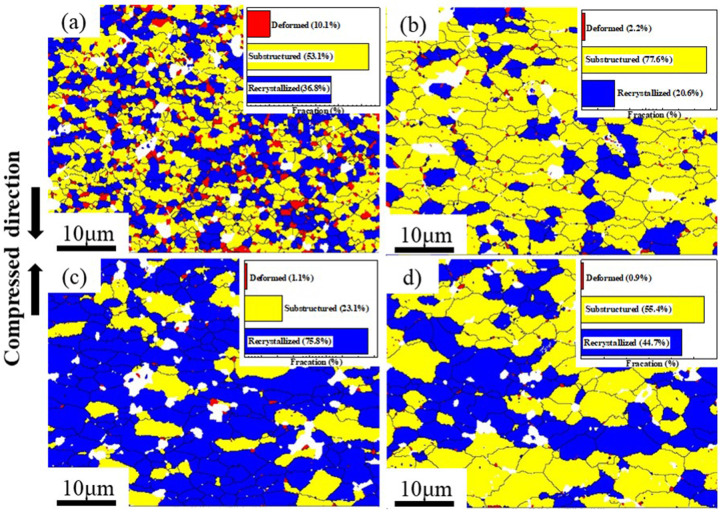
The recrystallized grains (blue parts), sub-grains (yellow parts) and deformed structure (red parts) in EBSD maps of samples compressed under different Z values: (**a**) lnZ = 30.0, (**b**) lnZ = 25.8, (**c**) lnZ = 21.7, (**d**) lnZ = 16.6 (HAGBs are in black lines, the inset figures are the percentages of recrystallized, substructured and deformed grain parts of the figures).

Zener-Hollomon parameters obtained from Equation (1) represent the combined effects of temperature and strain rate on deformation behavior. The change of Z values usually refer to microstructure evolution and the transformation of deformation mechanisms [13,37]. Figure 6 shows inverse pole figures (IPF) of the PM composites under different Z values. It can be seen that the grains in all deformation conditions are randomly oriented. At high Z parameter (e.g., lnZ = 30.0 at 300 °C/1 s^−1^), the majority of initial grains are elongated and perpendicular to compression direction as shown in the Figure 6a. Moreover, many fine equiaxed grains are formed on the initial grain boundaries. With decreasing Z values, the grains become larger and the shape of grains become more equiaxed, as shown in Figure 6b–d. Figure 7a–d show the grain size distribution of the PM composites in Figure 6. Apparently, the grain size is concentrated on a low value at high Z parameter. With decreasing the Z parameters, the peak value of grain size shifts to a larger value and the grains have a wider size distribution. Figure 7e compares the average grain size and aspect ratio of deformed microstructure with the initial states. The resultant grain sizes under all deformation conditions are always smaller than the initial ones. In particular, at high Z parameter, the average grains are remarkably refined due to hot deformation. The average aspect ratio of deformed samples are all larger than the initial ones. It firstly increases and subsequently decreases with the Z parameters declining. The change of grain size and aspect ratio are always closely related to the softening mechanisms under different deformation conditions. Figure 8 shows the recrystallized grains, sub-grains and deformed structures in the compressed composites under different Z parameters. It is evident that as the Z parameters decrease, the fraction of the deformed grains decreases. However, by decreasing the value of Z, the recrystallized fraction decreases first, followed by an increasing trend, and finally decreases again. It indicates that the deformation mechanisms change with decreasing the Z parameters.

## 4. Discussion

### 4.1. Deformation Mechanisms at Different Z Paramenters

**Figure 9:**
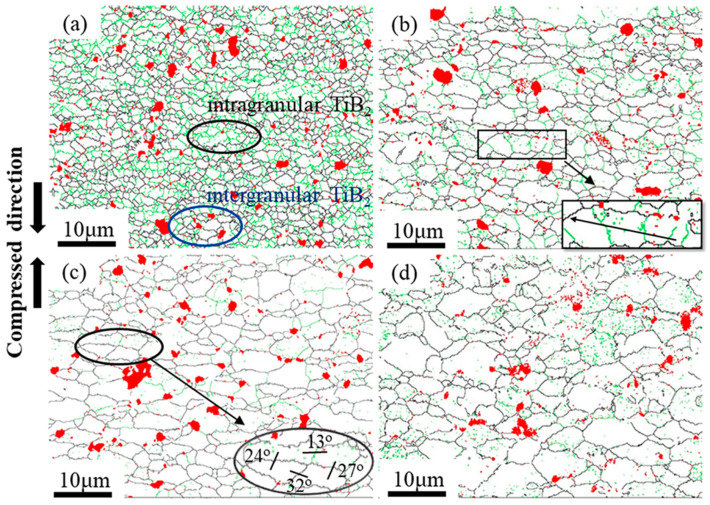
The grain boundary maps of samples under different Z values (**a**) lnZ = 30.0; (**b**) lnZ = 25.8; (**c**) lnZ = 21.7; (**d**) lnZ = 16.6 (the red phase is the TiB_2_ particle, low-angle grain boundaries (LAGBs) are in green lines, HAGBs are in black lines and the inset figures are enlarged images of the labled parts).

**Figure 10:**
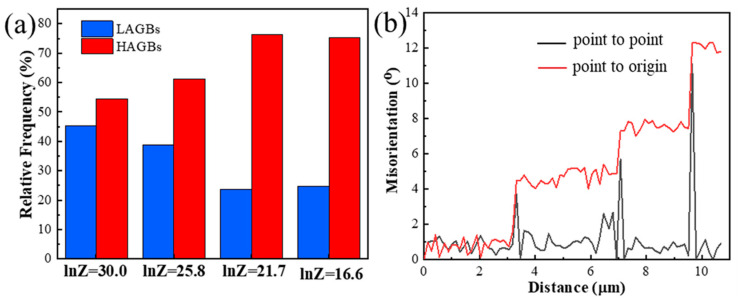
(**a**) The relative frequency of LAGBs and HAGBs of Figure 9, (**b**) the local (point to point) misorientation and cumulative (point to origin) misorientations along the black vector corresponding to Figure 9b.

The deformation behavior and microstructure evolution of the PM composites are generally decided by the related deformation mechanisms revealed by Z parameters. Figure 9 shows the grain boundaries maps obtained from EBSD of the PM composites. The relative frequency of LAGBs and HAGBs for PM composites of Figure 9 are shown in Figure 10a. At high Z parameter (lnZ = 30.0), it can be observed that a large number of subgrain structures are formed in the interior of grains (Figure 9a), and the fraction of LAGBs is high, up to 46% (Figure 10a). This is a typical DRV microstructure. Meanwhile, the LAGBs are formed around the intragranular TiB_2_ particles (black circle in Figure 9a), demonstrating that particles can change the misorientation of grain to promote the recrystallization [38]. Moreover, large amounts of fine recrystallization grains can be seen around intergranular TiB_2_ particles (blue circle in Figure 9a), which implies that high stored energy can be formed around particles to stimulate the grain nucleation (PSN). Therefore, the introduction of TiB_2_ particles can lead to the occurrence of DDRX.

At moderate Z values (lnZ = 25.8, lnZ = 21.7), the deformed grains are divided into several subgrains due to DRV, but the subgrains grow larger (Figure 9b,c) and the fraction of LAGBs decreases dramatically (Figure 10a) compared to those at high Z parameters. This trend suggests that the motion and arrangement of dislocations are strengthened. At the lnZ value of 25.8, the local misorientation and cumulative misorientations along the black vector of Figure 9b are shown in Figure 10b. The cumulative misorientations along the vector increase continuously and exceed 12°, which suggests that progressive subgrain rotation has been developed from the grain center to grain boundary [39,40]. When lnZ value reaches 21.7, the adjacent grains and subgrains are separated by low-misorientation grain boundaries, as shown by the black circle in Figure 9c. This phenomenon is suggested to result from the division of original grains, as recrystallization occurring with the dislocation density and grain misorientation rising. Generally, the continuous dynamic recrystallization (CDRX) mechanism will be promoted owing to the progressive subgrains rotation when the cumulative misorientations exceed 10–15° [41]. Therefore, the DRV and CDRX occur at moderate Z values.

At low Z value (lnZ = 16.6), the flow stress exhibits low values and the strain rate sensitivity *m* obtained from Appendix A (Equation (A3)) is about 0.53, which indicates the occurrence of superplastic deformation. At this deformation condition, the average aspect ratio of the grain is low (Figure 7e) and the fraction of recrystallization grains decreases (Figure 8), compared to the sample when lnZ = 21.7. Moreover, the subgrains in matrices disappear as shown in Figure 9d, while the relative frequency of LAGBs is not significantly changed (Figure 10a). This phenomenon suggests that the GBS may occur and make a positive impact on plastic deformation [42]. As well documented, the deformation at 0.001 s^−1^ is close to the creeping condition, and the strength of grain boundaries will be decreased at elevated temperature [43,44]. Under the diffusion creep mechanism, the HAGBs sliding may occur between the fine grains (<10 μm) to accommodate the dislocation sliding. At the early stage of superplastic deformation, the large grains undergo CDRX softening through the dislocation slip and climb, while the occurrence of GBS only locates in local regions [45]. As increasing the strain, the large grains could be refined and the deformation mechanism of GBS expands the whole sample. Thus, GBS and CDRX occur in this composite material at low Z parameters.

**Figure 11:**
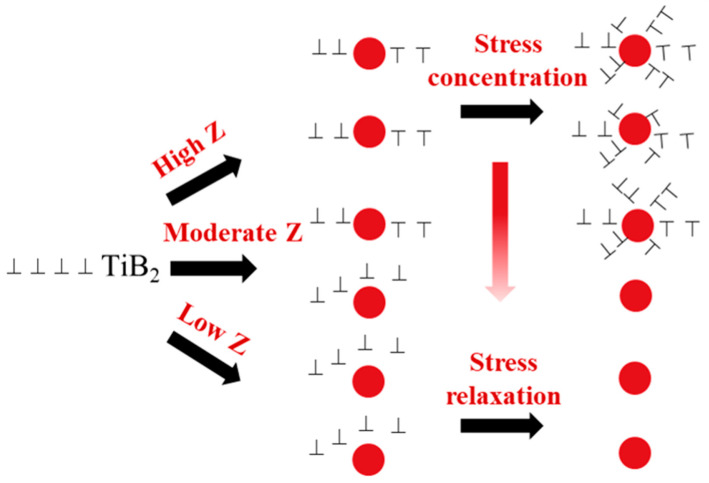
Schematic diagram of interactions of TiB_2_ particles and dislocations during hot deformation.

It is well known that ceramic particles hardly deform during the plastic deformation of PRAMCs, and thus a relatively high strain gradient exists near the TiB_2_–Al interface. Actually, the local high strain gradient is often accommodated by geometrically necessary dislocations [46]. Figure 11 shows a schematic diagram of interactions of TiB_2_ particles and dislocations during hot deformation. At high Z parameters, the TiB_2_ particles hinder the motion of dislocations. Thus, localized deformation zones with high stored energy and large misorientation can form around TiB_2_ particles, providing a large driving force and sites for PSN. At moderate Z values, the activation energy is enhanced at high temperatures and the rate of dislocation motion is small at low strain rates. The recrystallization can be triggered when the dislocation density around partial TiB_2_ particles reaches the critical level during gradual deformations. However, the stress concentration around partial TiB_2_ particles can be released by DRV, which hinders the initiation of PSN [47]. In the deformation process at low Z parameters, the climbing and diffusion of dislocation are enhanced, and thus dislocations can pass the particles to avoid accumulation and forming a dislocation wall [48].

From the discussion above, the softening mechanisms of different Z parameters are shown in Table 2. At high Z value, DDRX caused by PSN plays a dominant role in deformation, accompanied with the occurrence of DRV. With decreasing Z values, the PSN effects on DRX become weak and the DRV and CDRX are enhanced (Figure 8). The superplastic deformation occurs at low Z parameters and the dominant deformation mechanism is GBS.

### 4.2. Improved Workability of Powder Metallurgy (PM) Composites

As shown in the processing map (Figure 5), the PM composites exhibit huge stable working windows even at high Z parameters and have high power dissipation efficiency at low Z parameters. In contrast to PRAMCs fabricated by direct-chill casting, the PM composites exhibit excellent hot workability, which is closely related to the unique microstructure design.

#### 4.2.1. Safe Plastic Deformation at High Z Parameters

At high Z parameters, the materials are inclined to exhibit flow localization and deformation instability because the amount of heat energy generated during deformation cannot be dissipated at high strain rates. For the PRAMCs prepared by conventional casting technology, cracks are easily triggered in agglomerated particles to dissipate heat during deformation because of the low bonding strength [35]. Nonetheless, the PRAMCs fabricated by PM with dispersed particles have a more uniform deformation than the former, which can avoid particle fracture caused by stress concentration. Moreover, the dispersed particles can provide more nucleation sites for recrystallization. As shown in Figure 6a and Figure 8a, massive DRX grains caused by PSN are formed at high Z parameter, which can release the stress concentration around particles to prevent the occurrence of instability. On the other hand, the PM composites have fine initial grain size, which can give rise to a high fraction of grain boundaries and thus increase DRX kinetics to accelerate the onset of DRX during hot deformation [14,16,49]. In addition, the small grain size can reduce the migration distance of dislocations and subgrain boundaries, preventing the occurrence of local plastic flow [47]. As shown in Figure 9a, the fine deformed grains consist of only 2–3 subgrains, suggesting that the fine initial grains are beneficial for the motion of dislocations.

#### 4.2.2. Superplastic Deformation at Low Z Parameters

Generally, the superplastic deformation is related to the mechanism of GBS. It will occur when the grain size is less than 10 μm and the deformation temperature is higher than half of melting point (*T_m_*). Thus, the metallic materials with large initial grains (>10 μm) usually undergo DRX rather than GBS. The alloys with fine initial grains tend to coalesce during high-temperature deformations due to the enhancement of the atoms’ diffusion, causing the GBS severe difficulties geometrically. However, the PRAMCs with fine initial grain may exhibit superplastic deformation at low Z parameters. Wu et al. investigated the isothermal compress deformation of SiC/7075Al composites prepared by spray deposition and noted that the superplastic deformation occurred at 450 °C and the strain rate range of 0.001–0.1 s^−1^. At that deformation condition, the strain rate sensitivity was about 0.72 [36]. Actually, the PM composites fabricated in this study exhibit superplastic deformation at low Z values as well. The initial grain size of PM composites is less than 10 μm (Figure 1d), which is one of the necessary conditions for superplastic deformation. In addition, during hot deformation at low Z parameters, the large initial grains undergo DRX and can further refine the grain size (Figure 7e and Figure 8d), thanks to the pinning effect of dispersed TiB_2_ particles on grain growth. At low Z parameters, this stable microstructure leads to the occurrence of superplastic flow, which contributes to the excellent workability with the peak *η* value of 69%. Since the stress in superplastic deformation is lower than the preload of Gleeble-3180 equipment, the superplastic deformation is difficult to test at higher temperatures and lower strain rates. In the following research, we will study the superplasticity of PM composites in detail.

## 5. Conclusions

In this study, the deformation behavior and workability of the in-situ TiB_2_/7050Al composites fabricated by PM are investigated. The main conclusions can be summarized as follows:

(1) The constitutive equation based Arrhenius model is established as σp=10.0212ln{(Z5.93×109)13.64+[(Z5.93×109)23.64+1]12}. The activation energy is calculated as 143.11 kJ mol^−1^ approximately to the lattice self-diffusion energy of pure aluminum.

(2) The processing map of the PM composites based on DMM model is established at the true strain of 0.8. The PM composites exhibit excellent workability with wide safety-processing windows and the peak *η* is up to 69%.

(3) The Z parameters exert a significant influence on the microstructure evolution and deformation mechanism. At high Z value (lnZ = 30.0), the main softening mechanism is DDRX caused by PSN. At moderate Z values (lnZ = 25.8, lnZ = 21.7), the effects of PSN on recrystallization become weak and the dominant softening mechanisms transform into DRV and CDRX. At a low Z value (lnZ = 16.6), GBS occurs to promote the superplastic deformation.

(4) The uniform particle distribution and fine grain structures contribute to the good workability of the PM composites. The dispersed TiB_2_ particles promote the effects of PSN on recrystallization and inhibit the grain growth, and the initial fine grains are beneficial to the DRX and superplastic deformation.

## Figures and Tables

**Table 1:** The materials parameters of the PM composites.

Parameters	n_1_	β(MPa^−1^)	α(MPa^−1^)	*Q* (kJ/mol)	n	lnA
values	5.3028	0.0996	0.0212	143.11	3.64	22.50

**Table 2:** The softening mechanisms of the PM composites under different Z values.

Softening Mechanism	lnZ = 30.0	lnZ = 25.8	lnZ = 21.7	lnZ = 16.6
DDRX (PSN)	**√√**	**√**	**√**	**×**
DRV	**√**	**√√**	**√**	**×**
CDRX	**×**	**√**	**√√**	**√**
Superplastic flow (GBS)	**×**	**×**	**×**	**√√**

Note. **√√**: dominant mechanism, **√**: secondary mechanism, **×**: nonexistent mechanism.

## Nomenclature

The list of acronyms and symbols used in the article.

DDRX	discontinuous dynamic recrystallization	SEM	scanning electron microscope
CDRX	continuous dynamic recrystallization	EBSD	electron back-scattered diffraction
GBS	grain boundaries sliding	HAGBs	high angle grain boundaries
Z	Zener—Hollomon parameter	LAGBs	low angle grain boundaries
PRAMCs	particles reinforced aluminum matrix composites	Q	activation energy
PSN	particle-stimulated nucleation	DMM	Dynamic Material Model
DRX	dynamic recrystallization	*η*	power dissipation efficiency
PM	powder metallurgy	DRV	dynamic recovery
HIP	hot isostatic pressing	IPF	inverse pole figures

## Appendix A

In the DMM, the total power *P* of a workpiece transmitted by the tooling can be divided into two dissipater parts *G* and *J*. The part *G* represents dissipation through heat. The part *J* represents dissipation on metallurgical processes, such as DRX, DRV, superplastic deformation, or in the form of deformation damage. Thus,

(A1) $$P=\int_{0}^{\overset{\cdot }{\varepsilon }}\sigma d\overset{\cdot }{\varepsilon }+\int_{0}^{\sigma }\overset{\cdot }{\varepsilon }d\sigma =G+J$$

The part *G* and part *J* are related by the strain rate sensitivity *m*. Therefore, the *m* can be calculated by:

(A2) $$m=\frac{\partial J}{\partial G}=\frac{\overset{\cdot }{\varepsilon }\partial \sigma }{\sigma \partial \overset{\cdot }{\varepsilon }}=\frac{\overset{\cdot }{\varepsilon }\sigma \cdot \partial \log\sigma }{\sigma \overset{\cdot }{\varepsilon }\cdot \partial \log\overset{\cdot }{\varepsilon }}=\frac{\partial \log\sigma }{\partial \log\overset{\cdot }{\varepsilon }}$$

The efficiency of power dissipation, *η*, can be obtained from:

(A3) $$\eta =\frac{J}{J_{\max}}=\frac{2m}{m+1}$$

The occurrence of flow instability can be judged by the instability factor $\xi \left(\overset{\cdot }{\varepsilon }\right)$ and can be obtained by:

(A4) ξ(ε·)=∂ln(mm+1)∂ln(ε·)+m≤0

The variation of *η* with various temperatures and strain rates can establish a power dissipation map. The variations in the parameter $\xi \left(\overset{\cdot }{\varepsilon }\right)$ with temperatures and strain rates could structure an instability map which defines the flow instability where $\xi \left(\overset{\cdot }{\varepsilon }\right)\leq 0$.
